# Sequence-Independent DNA Adsorption on Few-Layered Oxygen-Functionalized Graphene Electrodes: An Electrochemical Study for Biosensing Application

**DOI:** 10.3390/bios11080273

**Published:** 2021-08-14

**Authors:** Narges Asefifeyzabadi, Torrey E. Holland, Poopalasingam Sivakumar, Saikat Talapatra, Ishani M. Senanayake, Boyd M. Goodson, Mohtashim H. Shamsi

**Affiliations:** 1 School of Chemical and Biomolecular Sciences, Southern Illinois University, 1245 Lincoln Drive, Carbondale, IL 62918, USA; narges.asefifeyzabadi@siu.edu (N.A.); ishani.senanayake@siu.edu (I.M.S.); bgoodson@chem.siu.edu (B.M.G.); 2 School of Physics and Applied Physics, Southern Illinois University, Carbondale, IL 62918, USA; torrey.holland@siu.edu (T.E.H.); psivakumar@siu.edu (P.S.); saikat@siu.edu (S.T.)

**Keywords:** DNA biosensors, graphene electrodes, inkjet-printing, trinucleotide repeats, label-free, electrochemical biosensors

## Abstract

DNA is strongly adsorbed on oxidized graphene surfaces in the presence of divalent cations. Here, we studied the effect of DNA adsorption on electrochemical charge transfer at few-layered, oxygen-functionalized graphene (GO_x_) electrodes. DNA adsorption on the inkjet-printed GO_x_ electrodes caused amplified current response from ferro/ferricyanide redox probe at concentration range 1 aM–10 nM in differential pulse voltammetry. We studied a number of variables that may affect the current response of the interface: sequence type, conformation, concentration, length, and ionic strength. Later, we showed a proof-of-concept DNA biosensing application, which is free from chemical immobilization of the probe and sensitive at attomolar concentration regime. We propose that GO_x_ electrodes promise a low-cost solution to fabricate a highly sensitive platform for label-free and chemisorption-free DNA biosensing.

## 1. Introduction

Graphene comprises a single layer of graphite in which carbon atoms arrange themselves in a 2D hexagonal lattice with metal-like charge carrier properties [1,2]. The interfacial properties of graphene interface, e.g., charge transport, can be easily and sensitively modulated by interactions with biomolecules [3,4,5], Therefore, single and multilayered graphene and its derivatives (i.e., graphene oxide and reduced graphene) have been widely exploited as sensing platforms to detect DNA [6,7,8], proteins [9,10,11,12], and small molecules [13] using a variety of detection methods including optical [6,13], scanning probe [10], electrical [7,8], and electrochemical techniques [9,11,12].

To harness the properties of such interfaces for sensitive biosensing platforms, interactions of nucleic acids with graphene-based surfaces have been extensively studied in two regimes, i.e., physisorption and chemisorption [14]. The interfacing of DNA and graphene surfaces is often achieved by the simple mixing of DNA oligonucleotide solution with graphene solution (a liquid/liquid interface) to form DNA-graphene hybrids [15,16], and DNA self-assembly on graphene electrodes (a liquid/solid interface) [17]. Theoretical studies have confirmed π stacking interactions between hydrophobic DNA basepairs and graphitic carbon rings of graphene as a driving force of adsorption [18,19,20]. Nucleobases show differential interactions where base rings are parallel to the graphene surface, which maximizes π–π stacking [21]. Theoretical studies have ranked the adsorption energies of bases on graphene as G > A > T > C [18,19], whereas experimental studies involving isothermal titration calorimetry indicates that the trend follows as G > A > C > T [22]. Thus, non-electrostatic interactions dominate the binding, where purine bases bind more strongly than pyrimidines [18,22,23]. Moreover, not all bases in a duplex conformation are adsorbed, and a diverse range of DNA conformations are likely to exist, which may result in competitive binding between bases and graphene oxide (GO)—leading to partial denaturation of double-stranded DNA (dsDNA) and exposition of single-stranded DNA (ssDNA) regions [24]. Both DNA and GO carry negative charge; therefore, a high ionic strength (up to a few mM Mg^2+^ or ≥100 mM NaCl) is used to facilitate the adsorption that overcomes the kinetic barrier [15]. DNA conformation can also influence the adsorption process due to intramolecular basepairing. A solution-based study involving the adsorption of ssDNA on GO has revealed that shorter DNA adsorbs faster and tighter to the surface, whereas lower pH and higher ionic strength conditions favor the adsorption. Despite the expanded theoretical and experimental research in this area, the question of how DNA interaction with graphene-based materials such as oxidized graphene surfaces can modulate electrical and electrochemical charge transport across the interface—a liquid/solid interface—is underexplored.

**Scheme 1:**
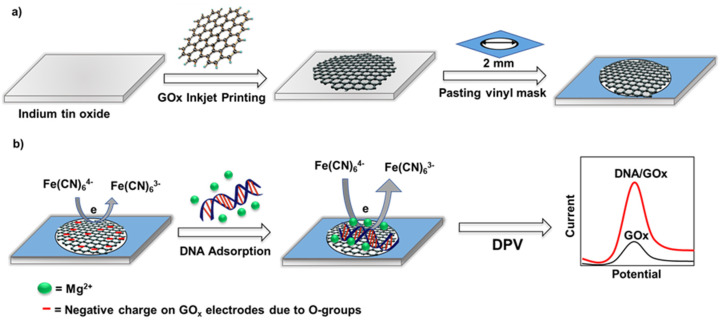
(**a**) Illustrating fabrication of electrodes by inkjet printing of exfoliated few-layered oxygen functionalized graphene (GO_x_) on indium tin oxide substrate, followed by adhesion of a vinyl mask selectively exposing a GO_x_ surface with 2 mm diameter. (**b**) Depiction of sensing mechanism: the lower current on the unmodified electrode is due to the repulsion between the negatively charged redox probe and the negative O-groups on the GO_x_ surface. Adsorption of DNA sequence on a GO_x_ surface in presence of Mg^2+^ ions reduces the electrostatic repulsion, which leads to higher diffusion of the redox probe producing higher electrochemical current measured by DPV.

For this study, we hypothesized that DNA adsorption on oxidized graphene interface may lead to unique electrochemical signatures, which eventually may lead to development of a simple electrochemical DNA biosensor. First, we prepared and thoroughly characterized a few-layered, oxygen-functionalized graphene ink (GO_x_), followed by fabricating the GO_x_ electrodes on indium tin oxide (ITO) surfaces by inkjet printing, as illustrated in Scheme 1a. Then, we physisorbed the DNA sequences on the GO_x_ electrodes in high divalent cation concentration (20 mM Mg^2+^), and monitored the electrochemical responses of the interface using differential pulse voltammetry, as depicted in Scheme 1b. The proposed sensing mechanism in the scheme explains the lower current obtained on the unmodified electrode due to repulsion between the negatively charged redox probe and negative O-groups on the GO_x_ surface. Then, the adsorption of the DNA sequence on a GO_x_ surface in presence of Mg^2+^ ions reduces the electrostatic repulsion, which leads to higher diffusion of the redox probe producing higher electrochemical current measured by DPV. In particular, we studied their electrochemical signature with respect to sequence type, conformation, concentration, length, and ionic strength in the presence of a soluble redox probe, Fe(CN)_6_^3−/4−^. Finally, we performed a proof-of-concept biosensing test that does not require chemical labelling and immobilization of the probe.

## 2. Experimental

### 2.1. Materials

For the sequence type, trinucleotide repeats sequences (TNRs) were used with CGG, CAG, and GAA repeats. All the synthetic TNR oligos were purchased from Integrated DNA Technologies (Coralville, IA, USA). The different sequences and lengths of the TNRs are shown in Table S1. Graphite flakes, 99% carbon basis (−325 mesh particle size, ≥99%), ethyl cellulose with viscosity 4 cP (5%) in toluene/ethanol 80:20 (lit), and 48% ethoxyl, ɑ-Terpineol (90%), tris(hydroxymethyl)aminomethane (Tris-ClO_4_) were purchased from Sigma-Aldrich (St Louis, MO, USA). Ethanol (95%); cyclohexanone, K_4_[Fe(CN)_6_], K_3_[Fe(CN)_6_] (99+%), and phosphate-buffered saline (PBS) pH 7.4 (5× solution) were purchased from Fisher Scientific. Holey carbon-coated grids for electron microscopic studies were purchased from SPI Supplies (West Chester, PA, USA). Indium tin oxide coated substrate (ITO) with resistivity 4–8 Ω/sq was obtained from Delta Technologies (Loveland, CO, USA). An Electrochemical Workstation CHI 660E from CHI Instruments (Austin, TX, USA) was used for electrochemical characterization of the interface. A Metrohm Autolab potentiostat was used for differential pulse voltammetry measurements. An Ag/AgCl (with KCl solution) reference electrode and platinum wire auxiliary electrode was procured from Bioanalytical Systems Inc. (West Lafayette, IN, USA). Fujifilm Dimatix Materials Printer (DMP-2800) was used for printing on ITO substrate and purchased from Integrity Industrial Inkjet Integration (W. Lebanon, NH, USA). A NanoDrop One Spectrometer from Thermo Scientific was used for UV-Visible characterization of the few-layered graphene ink. A Graphtec Cutting Plotter (CE6000-40) was used to cut vinyl sheets to be used for covering the ITO substrates, while exposing a 2 mm diameter circle for studying the interface. Open-source software, Inkscape (Version 0.91), was used to design patterns for printing. A portable four-point probe test meter (HM21) from Jandel Engineering Limited (UK) was used for electrical characterization of the printed graphene ink. Raman characterization of graphene ink was performed using a Horiba iHR550 imaging spectrometer with near-infrared (NIR) excitation light source at a wavelength of ~785 nm (iBeam-Smalt-785-S-WS, TOPTICA Photonics). A Hitachi H-7650 transmission electron microscope operating at 60 kV and a Quanta 450 FEG (FEI) at the SIU Imaging Center were used for electron microscopic characterization of graphene ink and fabricated devices. Dynamic Light Scattering (DLS) measurements were performed using a DynaPro NanoStar purchased from Wyatt Technology Corporation.

### 2.2. Preparation of Ink

First, graphene sheets were exfoliated by ultrasonic exfoliation of graphite flakes in ethyl cellulose/ethanol mixture using an ultrasonication method as reported earlier [25,26], which gives a solid product of graphene/ethyl cellulose (Gr/EC). To prepare the graphene-based ink for inkjet printing, the Gr/EC powder was dispersed in an 83:17 cyclohexanone/terpineol mixture, sonicated for 2.5 h [25,26], and stored at room temperature. For inkjet printing, 3.5% ink concentration was prepared having 10 cP viscosity, which is suitable for inkjet printing using Dimatix printer.

### 2.3. Characterization of Ink

For ink characterization, a UV-vis spectrum scan of the ink was obtained between 200–800 nm by dropping a 5 μL aliquot of the ink on the NanoDrop Spectrometer. Transmission electron microscopic (TEM) images of the graphene nanosheets in the ink were obtained by dropping a small amount of the ink on a holey carbon coated grid followed by air drying before imaging. To measure the particle size of the ink, 200 µL of the sample was diluted up to 50 mL using absolute ethanol as a solvent. The mixture was sonicated for one minute. The DLS analysis was performed using 300 µL of the solution. This dilution factor allowed a mostly translucent solution to be analyzed. To confirm the type of graphene material, the ink was analyzed by Raman spectroscopy after annealing at 350 °C. The ink was directly analyzed on glass slides (Eisco microscope slides) using a modular Raman microscope system. A grating of 600 g/mm was used in the spectrometer, and a CCD camera on the Raman microscope was used to locate the ink deposition. After performing the characterization tools mentioned above, we identified our ink as few-layered oxygen functionalized graphene (GO_x_).

### 2.4. Fabrication and Characterization of GO_x_ Electrodes

The GO_x_ ink was printed on ITO substrates using a Dimatix inkjet printer, employing parameters as previously reported [26]. For resistance measurements, line patterns were printed on ITO using printing cycles (3, 5, and 7) and sintered in a furnace for 30 min at 350 °C. Later, thicknesses of the patterned lines from different cycles were measured by scanning electron microscopy (SEM) before and after sintering.

Circular graphene patterns 2 mm in diameter were printed on the clean and dry ITO substrates for DNA adsorption studies. Following printing, thermal annealing of the electrodes was performed at various temperatures (250 °C to 350 °C) in air for 30 min. The working electrode area was separated from the rest of the ITO surface by pasting a vinyl sheet on the surface with a 2 mm-diameter hole to expose the inkjet-printed electrode while covering the rest of the electrically conducting area. The electrochemical impedance spectroscopy (EIS) was performed in the presence of 1 mM K_4_[Fe(CN)_6_]/K_3_[Fe(CN)_6_] (1:1) prepared in PBS buffer (pH 7.2) at 0.3 V against Ag/AgCl reference (3 M KCl) using 5 mV amplitude and 100 kHz to 0.1 Hz frequency. Then, cyclic voltammetry with a potential window of 0−0.6 V was performed at the scan rates of 50–500 mV/s in 1 mM K_4_[Fe(CN)_6_]/K_3_[Fe(CN)_6_] (1:1) prepared in PBS buffer (pH 7.2). The electrochemical measurements were recorded against an Ag/AgCl reference electrode and a platinum wire counter electrode.

### 2.5. Electrochemical Study of DNA/GO_x_ Interface

Solution hybridized double-stranded DNA (dsDNA) were prepared in 100 µM Tris buffer (pH 8.5) containing 200 mM NaCl and 20 mM MgCl_2_. The mixture was heated to the melting temperature for 30 min and annealed at room temperature for 1 h. For electrochemical characterization of the DNA/GO_x_ interface, 5 µL aliquots of DNA solution were dropped on the GO_x_ electrode and incubated for 12–16 h at 4 °C. For concentration-dependence study, a range from 1 aM to 10 nM was used to adsorb dsDNA (dsCGG-8, dsCAG-8, and dsGAA-8; see Table S1) on the GO_x_ surface. For the length–dependence study, 5, 8, and 10 repeat lengths of 1 aM dsCGG were adsorbed on the GO_x_ surface. For surface hybridization detection, concentration of the physisorbed probe sequence (ssCGG-8) was first optimized, and later exposed to complementary GGC-8 target and noncomplementary sequences (NC-1 = TTC-8 and NC-2 = CAG-8) at 1 aM concentration. To confirm the ionic strength effect on the adsorption, EDTA was added to the DNA solution to chelate Mg^2+^ before the adsorption on the GO_x_ surface. Following the adsorption of the sequences for various variables, differential pulse voltammetry was performed with a potential range of 0.1–0.5 V and an amplitude of 0.05 V in 1 mM Fe(CN)_6_^3−/4−^ prepared in PBS buffer (pH 7.2).

## 3. Results and Discussion

**Figure 1:**
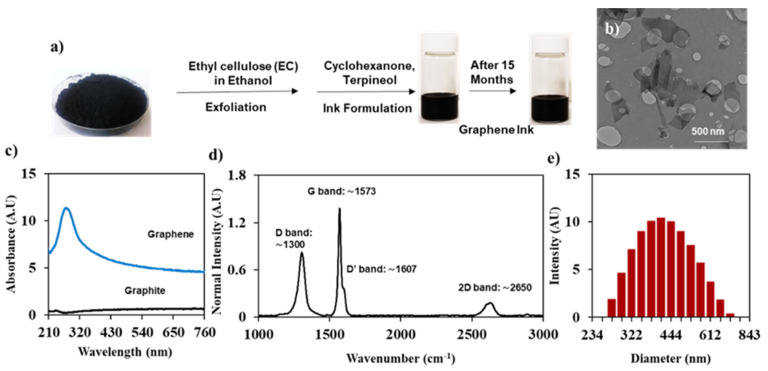
Synthesis and characterization of the few-layered graphene ink. (**a**) Exfoliation and formulation, (**b**) TEM image of exfoliated graphene sheets, (**c**) UV−vis spectrum of graphite and graphene ink, (**d**) Raman spectra of the ink, and (**e**) dynamic light scattering of the ink.

Figure 1a illustrates the graphene-based ink preparation in an environmentally benign solvent as reported previously [25,26,27], which was found stable even after 15 months of storage at room temperature. The graphene-based ink was characterized by TEM, as shown in Figure 1b, indicating that the process yielded few-layered graphene nanosheets. The UV-vis characterization shown in Figure 1c confirms the formation of graphene sheets with a strong absorption peak at 275 nm due to excitation of π-plasmon of graphitic structure, which corroborates the previous reports [28,29,30]. The bands shown in the Raman spectrum in Figure 1d are similar to annealed exfoliated graphene comprising four bands, i.e., G band at ∼1590 cm^−1^, 2D band at ∼2700 cm^−1^, and disorder-related D and D’ peaks at ∼1350 and ∼1620 cm^−1^, respectively [27]. The sharpness of the D and G bands are similar to the graphene-ethylene cellulose nanocomposite [27]. As previously indicated, a lower value of the I(D)/I(G) peak ratio suggests an increase in graphitization and lower defects, which can lead to better electrical conductivity [31]. Moreover, defects and oxidation might be introduced in the material during the sonication and annealing process, and may also arise from the smaller sizes of the sheets [27,32]. The I(D)/I(G) value for this ink is ∼0.58, which indicates a moderate level of defects and oxidation during exfoliation and annealing [27], while the sharp peaks confirm the improved graphitization compared to typical graphene oxide (GO) material [33]. The composition of the ink was verified by EDS elemental analysis (Figure S1 in Supplementary Information) showing 56% C-atoms and 34% O-atoms after sintering the surface yielding a C/O ratio = 1.5, which confirms the partial oxidation of the ink during the formulation and annealing process. Figure 1e shows the particle size distribution of 82% of the particles with a diameter in the range of 250 to 700 nm, with an average diameter of 432 nm. The extensive characterization presented here suggests the ink material as few-layered oxygen-functionalized graphene ink (GO_x_), where the presence of a moderate amount of oxygen functional groups on the surface may facilitate intermediate loadings and binding interactions for DNA [34].

**Figure 2:**
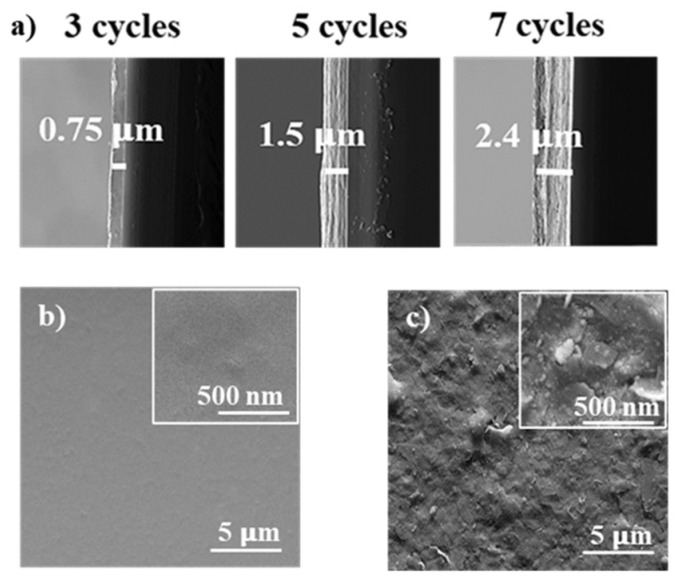
(**a**) SEM images of 3, 5, and 7 printed cycles of GO_x_ ink after sintering at 300 °C. (**b**) SEM images of top surface of the printed GO_x_ electrode before sintering and (**c**) after sintering.

**Figure 3:**
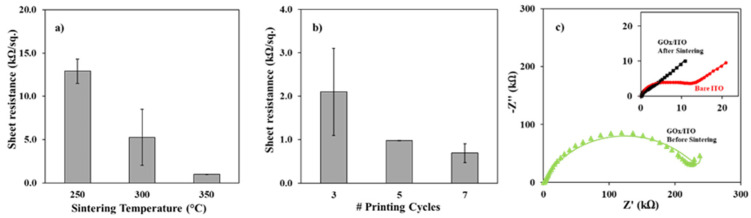
(**a**) Resistivity versus sintering temperature for 30 min following five printing cycles. (**b**) Resistivity versus number of printing cycles following sintering under fixed conditions (350 °C for 30 min). (**c**) Nyquist form of the EIS plot for bare ITO and GO_x_/ITO (before and after sintering).

The GO_x_ ink was printed and characterized by various methods. We investigated the relationship between the printing cycles and pattern thickness by SEM shown in Figure 2. The cross-section images of 3, 5, and 7 printing cycles of GO_x_ patterns following sintering at 300 °C (Figure 2a) confirm the increase in thickness of the printed pattern with the number of printing cycles, i.e., 3 cycles (0.75 µm), 5 cycles (1.5 µm), and 7 cycles (2.4 µm). The morphology of the GO_x_ surface reveals a smooth surface before sintering, and the appearance of wrinkles after sintering. Such wrinkled roughness may endow a material with higher electrochemical current due to an increase in the surface area, as previously reported [35,36]. Then, we investigated the electrical and electrochemical behavior of the GO_x_ electrode. Figure 3a shows that the resistivity significantly decreased with high precision when the sintering temperature reached at 350 °C. Figure 3b indicates that the trend decreases with the printing cycles, where five cycles have significantly higher precision. Figure 3c shows the Nyquist form of electrochemical impedance spectroscopy (EIS) plots for the bare ITO substrate (see inset), GO_x_ ink before sintering, and GO_x_ ink after sintering (see inset). The charge transfer resistance of the GO_x_ ink before sintering (R_ct_ = 246 kΩ) is almost two orders of magnitude higher than that of bare ITO (R_ct_ = 9.5 kΩ). While the charge transfer resistance of the GO_x_ ink following sintering decreased (R_ct_ = 4.2 kΩ), which is less than 50% of the bare ITO resistance. This is an indication of substantially improved electrochemical property of the GO_x_/ITO electrode after sintering. The modified Randle’s equivalent circuit model used for fitting the Nyquist plot and the fitting values are shown in Figure S2 and Table S2, respectively. Following this characterization, we used the conditions of five printing cycles and sintering at 350 °C for 30 min to study the DNA/GO_x_ interface.

**Figure 4:**
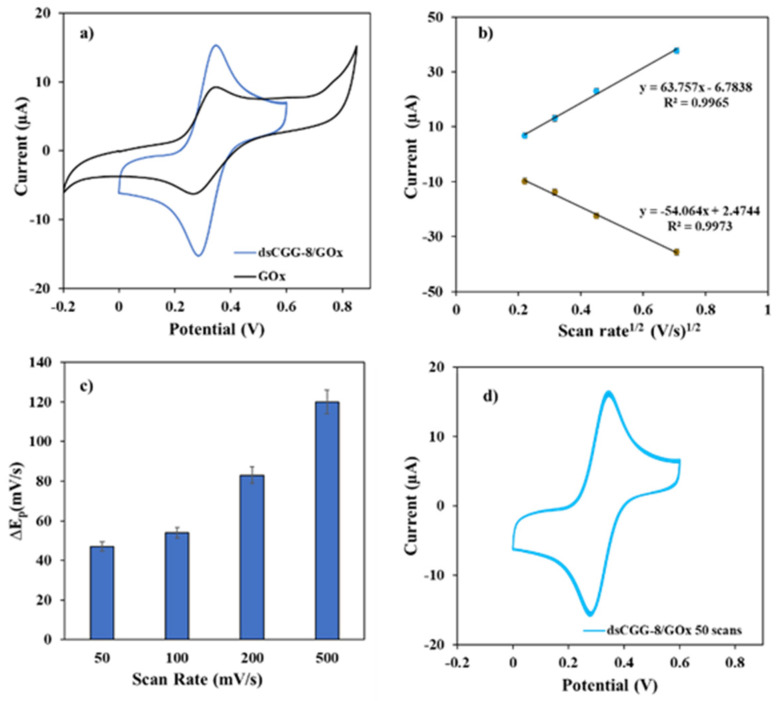
(**a**) Cyclic voltametric curves before and after adsorption of dsCGG-8 on GO_x_. (**b**) Plot of cathodic and anodic voltametric current of dsCGG-8 as a function of square root of scan rate. (**c**) Cathodic–Anodic peaks separation (∆E_p_) versus scan rate for the dsCGG-8 modified electrodes. (**d**) 50 scans of CV curves of dsCGG-8 on GO_x_ electrode. The electrochemical measurements were performed in presence of 1 mM Fe(CN)_6_^3−/4−^ using Pt wire counter electrode and Ag/AgCl (3 M KCl) reference electrode. Scan rate for CV measurement is 100 mV/s. Concentration of dsCGG-8 was 10 nM.

**Figure 5:**
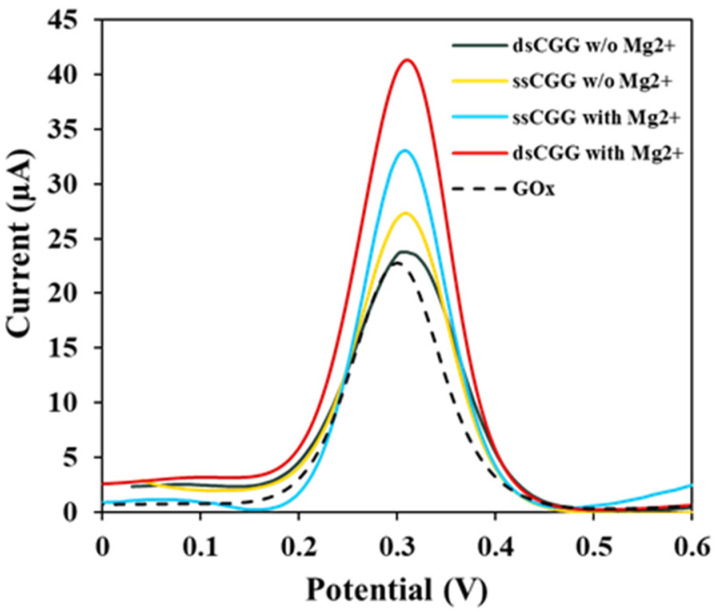
DPV curves of unmodified GO_x_ and after exposure to 10 nM ssCGG-8 and dsCGG-8 in presence (with) and absence (w/o) of Mg^2+^.

The electrochemical current of the surface-adsorbed dsCGG-8 (10 nM) was investigated on the GO_x_ electrode by voltametric techniques. Figure 4a shows cyclic voltametric curves before and after DNA modification of the GO_x_ electrode. The integrated peak current of the dsCGG-8/GO_x_ (12.5 µA) is almost twice the GO_x_ current (6.5 µA). The enhanced charge transport behavior of the DNA modified GO_x_ electrode was monitored over a range of 50–500 mV/s scan rates (Figure 4b), which follows the Randles–Sevcik equation model wherein peak current is proportional to square root of the scan rate [37]. However, Figure 4c shows that cathodic-anodic peaks separation (∆E_p_) increases with the scan rate (see cyclic voltamogram in Figure S3), which is an indicative of a quasi-reversible electron kinetics at the electrode surface and is typical to the DNA modified electrodes [38]. Figure 4d shows the cyclic voltammetry performed up to 50 scans for the DNA modified GO_x_ electrode, which presents no substantial change in the peak current and overpotential with the number of scans, thus it suggests a stable DNA/GO_x_ interface. We attribute this strong adsorption affinity to mild oxidation of the graphene sheets in the GO_x_ ink (C/O = 1.5), similar to recently reported strong adsorption of ssDNA and dsDNA on graphdiyne surface with a low oxidation degree [39]. Moreover, high cationic strength screens negative charge between the two materials and facilitates the adsorption. There is an intriguing question of whether this current enhancement is due to the DNA mediated electron transport or enhanced diffusion of the redox probe owing to high cationic strength environment. To test the effect, we added EDTA in the DNA solutions to chelate the Mg^2+^, followed by incubation of the mixture with the GO_x_ electrode. Figure 5 shows the DPV current of ssCGG-8 and dsCGG-8 in the presence and absence of Mg^2+^ and compared with the unmodified GO_x_ electrode current. Evidently, the current in the absence of Mg^2+^ is very similar to background current or unmodified GO_x_ current (~22 µA). This result confirms the dual role of Mg^2+^, i.e., promoting the DNA adsorption as suggested previously [15], and reducing the electrostatic repulsion between the redox probe and the electrode surface. It is important to note that DNA modified electrodes have shown higher resistance to charge transfer than that of unmodified electrode in low cationic strength due to lack of diffusion of the redox probe [40], which was improved after the addition of divalent cations [41,42,43]. Based on these results, we conclude that the high concentration of divalent cations in the environment screen the negative charge on both materials, which ultimately increases the diffusion of the redox probe, Fe(CN)_6_^3−/4−^, to the electrode surface leading to higher current response.

**Figure 6:**
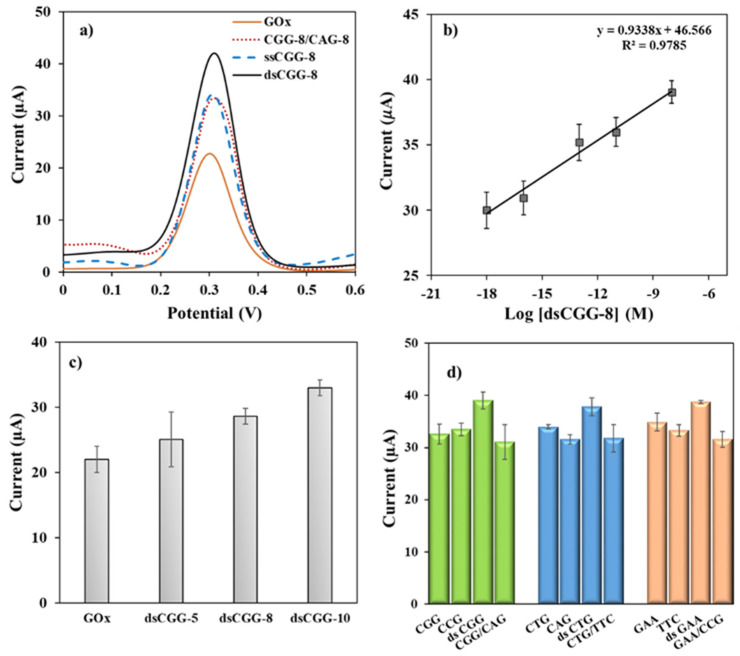
(**a**) Differential pulse voltamogram of 10 nM ssCGG-8, dsCGG-8, and noncomplementary mixture CGG-8/CAG-8. (**b**) Calibration curve dsCGG-8. (**c**) DPV current response of various lengths of dsCGG at 1 aM concentration. (**d**) DPV current response of various single-stranded, double-stranded, and noncomplementary sequences at 10 nM concentration. Error bars represent standard deviation for N ≥ 3.

Next, we tested the current responses of conformation, concentration, length, and sequence types of DNA using DPV. Figure 6a shows the DPV curves of GO_x_, ssCGG-8, dsCGG-8, and a noncomplementary mixture of CGG-8/CAG-8 at 10 nM concentration. The current response of the double-stranded conformation (dsCGG-8) was higher than the single-stranded conformation (ssCGG-8), while the current response of the noncomplementary mixture showed similar current response as the single-stranded conformation, which corroborates the fact that noncomplementary strands remained unhybridized and in single-stranded conformation. Figure 6b shows the current response of dsCGG-8 over a range of 1 aM to 10 nM. The least-squares regression for dsCGG-8 formed a linear curve fit with a correlation coefficient R^2^ = 0.9785 (Figure 6b). The detection limit signal, LOD*_y_*, was obtained using LOD*_y_* = (blank signal + 3*SD*_blank_*) − intercept; where blank signal = 22 µA, 3*SD*_blank_* = 3*2.5 µA, and intercept = 46.566 µA. Then, the ‘theoretical’ limit of detection concentration, LOD*_x_*, was determined as 0.8 aM by calculating antilog (LOD*_y_* ÷ sensitivity), where sensitivity or the slope of the curve ‘m’ is 0.9338. Figure 6c shows the current responses of dsCGG comprising 5, 8, and 10 dsCGG repeat units (i.e., 15, 24, and 30 nucleotide lengths respectively) at 1 aM concentration regime. The upward trend of the current response with respect to length indicates that the increase in DNA adsorption aided by Mg^2+^ facilitates the charge transport at the interface. This length-dependent current response may lead to sensitive and label-free discrimination of normal and abnormal lengths of DNA repeat sequences associated with neurodegenerative diseases, as reported recently on MoS_2_ nanosheets surfaces [44]. Then, we tested the adsorption of various single-stranded, double-stranded, and noncomplementary sequences of same length (i.e., 8 trinucleotide repeats or 24 nucleotides) at 10 nM concentration. Figure 6d shows the current responses of trinucleotide repeat types CGG, CAG, and GAA (single-, double-, and noncomplementary) revealing the following trends. First, the current responses of double-stranded conformations, in all cases, are higher than their single strands. Second, the noncomplementary responses are similar to single-stranded conformation. Third, the current responses for all sequences are similar with respect to their conformation. Overall, we learn that the increase in DNA interaction with the surface in the high cationic environment increases the current response, which can be indicative of its conformation, concentration, and length. Despite the differential adsorption affinity of graphene-based materials for different nucleotide types, the DNA adsorption was sequence-independent. This result can be rationalized as the effect of high concentration of Mg^2+^ perhaps masks the sequence-dependent affinity between DNA and GO_x_.

**Figure 7:**
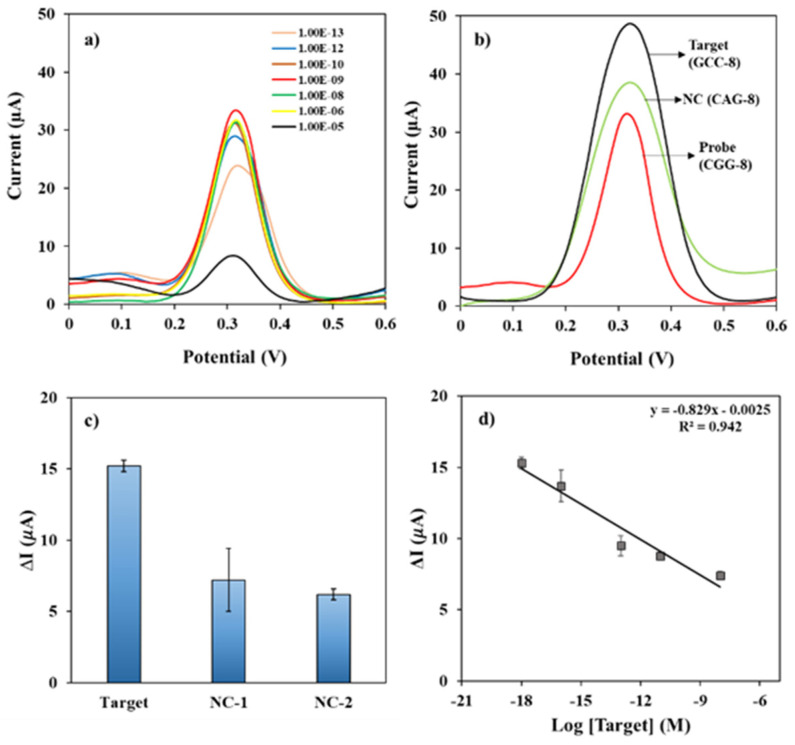
DPV current of (**a**) different concentration of probe sequence (ssCGG-8), (**b**) 1 nM probe, surface hybridized target (GGC-8) and noncomplementary (CAG-8) at 1 aM concentration. (**c**) Difference in current ∆I (target current − probe current) for complementary (CCG-8) and noncomplementary targets (NC-1 = TTC-8 and NC-2 = CAG-8), and (**d**) ∆I versus concentration of the surface hybridized CCG-8 [Target]. Hybridization time 1 h.

Finally, we tested the performance of the interface for biosensing application. We optimized the adsorption of the probe sequence, ssCGG-8, which was later exposed to a complementary target and two different noncomplementary sequences. Figure 7a shows the response of ssCGG-8 probe sequence between 0.1 pM-10 µM concentrations. The maximum current of 32.3 ± 1.2 µA was observed at 1 nM concentration, which was assumed to be saturation of the surface with the probe strands. The current responses in Figure 7b show significant enhancement in the current following the surface hybridization reaction with the complementary target (CCG-8) at 1 aM concentration. The change in current ∆I, which is (target current−probe current), shown in Figure 7c, evidently distinguishes between the complementary target and the noncomplementary sequences (NC-1 = TTC-8 and NC-2 = CAG-8). One may notice that the current enhancement of surface hybridized complex is much higher than the solution hybridized complex at this concentration regime, which intrigued us to study the effect of target concentration, as shown in Figure 7d. Although current was enhanced following hybridization over a wide concentration range of the target, ∆I shows a downward trend with the target concentration. As mentioned above, competitive binding between nucleobases and graphene oxide (GO) exist, leading to partial denaturation of double-stranded DNA [24]. Based on this knowledge, we rationalize that not all target strands will hybridize on the surface, and unhybridized target strands may adsorb non-specifically on the pre-saturated interface, leading to a decrease in current by impeding diffusion of the redox probe. Nevertheless, further investigation is necessary to rationalize the high sensitivity at very low target concentration and the downward concentration trend. We propose that, in future applications, the strong adsorption of the probe in the presence of Mg^2+^ and intense surface hybridization signal would eliminate the steps involved in covalent immobilization of a probe (chemisorption) [14] or expensive modification of the probe with 1-pyrenebutanoic acid succinimidyl ester (PASE) linker for stronger π–π interactions with graphene surface [45]. In contrast to other electrochemical detection of TNRs [46,47], this label-free GO_x_ platform does not rely on guanine oxidation for sensing—therefore, a noninvasive method—and shows higher sensitivity down to the attomolar level compared to a recently reported solution-gated graphene transistor having detection limit only up to femtomolar range.

## 4. Conclusions

To best of our knowledge, this is the first comprehensive study on the electrochemical behavior of the DNA adsorbed, oxidized graphene electrodes (GO_x_). The GO_x_ was synthesized and thoroughly characterized followed by fabrication of the GO_x_ electrodes on ITO surfaces. The DNA adsorption on the GO_x_ electrodes was performed in the high ionic strength (20 mM Mg^2+^ + 200 mM Na^+^). The DNA-adsorbed GO_x_ surfaces produced significantly higher current due to enhanced diffusion of the soluble redox probe, which was rationalized as the effect of high concentration of divalent cation (Mg^2+^) in the environment. The study reveals that increase in DNA interaction with the surface increase the current response which can be indicative of its conformation, concentration, and length. The detection limit of the dsCGG adsorption on the GO_x_ was 0.8 aM. The sequence-independent behavior was reasoned as the effect of high concentration of Mg^2+^ that neutralizes the negative charges on both materials and facilitates the adsorption of different types of sequences. Surface hybridization between the complementary sequences was distinguishable from the noncomplementary sequence, even at 1 aM concentration regime. Nevertheless, further study is suggested to investigate the difference in concentration trends of solution versus surface hybridization. We propose that the strong DNA adsorption in presence of Mg^2+^ and intense surface hybridization signal can develop into simple and label-free DNA biosensors on inkjet-printed GO_x_ devices.

## Data Availability

Not applicable.

## Supplementary Materials

The following are available online at https://www.mdpi.com/article/10.3390/bios11080273/s1.
