# Supplementary material for: Sequence-Independent DNA Adsorption on Few-Layered Oxygen-Functionalized Graphene Electrodes: An Electrochemical Study for Biosensing Application
Source: Biosensors (Basel). 2021 Aug 14;11(8):273. doi: 10.3390/bios11080273 (PMC8394360; doi:10.3390/bios11080273)
Supplement: Supplementary file 1 [file biosensors-11-00273-s001.zip › biosensors-1335527-supplementary.pdf]

TNR-GOx-3

Supplementary Information

Sequence-Independent DNA Adsorption on Few-Layered Oxygen-Functionalized Graphene Electrodes: An Electrochemical Study for Biosensing Application

Narges Asefifeyzabadi [1], Torrey E. Holland [2], Poopalasingam Sivakumar [2], Saikat Talapatra [2], Ishani M. Senanayake [1], Boyd M. Goodson [1] and Mohtashim H. Shamsi [1,*]

[1] School of Chemical and Biomolecular Sciences, Southern Illinois University, 1245 Lincoln Drive, Carbondale, IL 62918, USA; narges.asefifeyzabadi@siu.edu (N.A.); ishani.senanayake@siu.edu (I.M.S.); bgoodson@chem.siu.edu (B.M.G.)

[2] School of Physics and Applied Physics, Southern Illinois University, Carbondale, IL 62918, USA; tor-rey.holland@siu.edu (T.E.H.); psivakumar@siu.edu (P.S.); saikat@siu.edu (S.T.)

* Correspondence: mshamsi@siu.edu

Table S1. Single-stranded and double-stranded DNA sequences.

TNR type	Single-Stranded Sequence	#Repeats
ssCGG-8	5'-CGG CGG CGG CGG CGG CGG CGG CGG -3'	8
ssCCG-8	5'-CCG CCG CCG CCG CCG CCG CCG CCG -3'	8
ssGAA-8	5'-GAA GAA GAA GAA GAA GAA GAA GAA -3'	8
ssTTC-8	5'-TTC TTC TTC TTC TTC TTC TTC TTC-3'	8
ssCTG-8	5'-CTG CTG CTG CTG CTG CTG CTG CTG -3'	8
ssCAG-8	5'- CAG CAG CAG CAG CAG CAG CAG CAG -3'	8
	**Double-Stranded Sequence**	
dsCGG-5	5'-CGG CGG CGG CGG CGG -3'<br>3'- GCC GCC GCC GCC GCC -5'	5
dsCGG-8	5'-CGG CGG CGG CGG CGG CGG CGG CGG -3'<br>3'- GCC GCC GCC GCC GCC GCC GCC GCC -5'	8
dsCGG-10	5'-CGG CGG CGG CGG CGG CGG CGG CGG CGG CGG -3'<br>3'- GCC GCC GCC GCC GCC GCC GCC GCC GCC GCC -5'	10
dsCTG-8	5'-CTG CTG CTG CTG CTG CTG CTG CTG -3'<br>3'- GAC GAC GAC GAC GAC GAC GAC GAC -5'	8
dsGAA-8	5'-GAA GAA GAA GAA GAA GAA GAA GAA -3'<br>3'-CTT CTT CTT CTT CTT CTT CTT CTT -5'	8

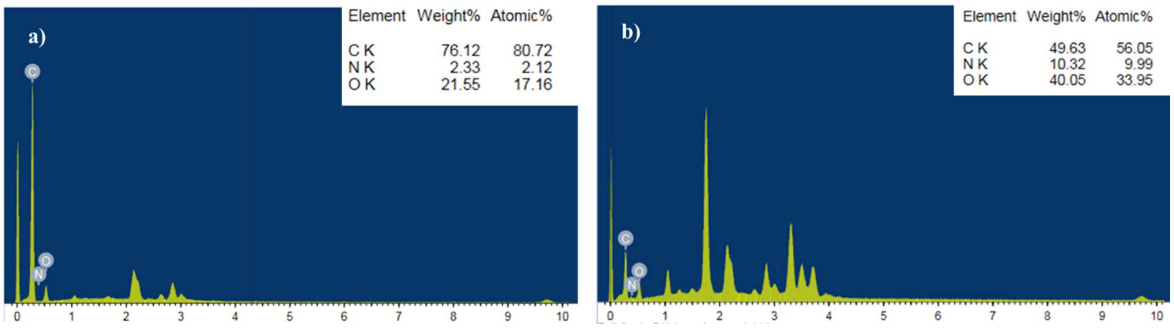

Figure S1. EDS analysis of printed Gr on ITO a) before and b) after sintering.

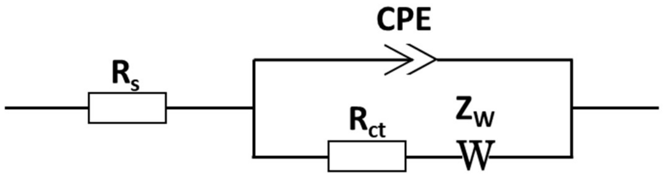

Figure S2. Randle's equivalent circuit elements.

Table S2. EIS extracted parameters of **Figure 2c**. The average values with standard error of modified Randle's equivalent circuit elements. The errors were calculated for N ≥ 4 separate measurements.

	**R_s kΩ**	**C_{dl} μF (10^{-2})**	**R_{ct} kΩ**	**W kΩ**
Bare ITO	0.2 (0.002)	99.9 (3.10)	9.5 (0.1)	0.005 (1.1) × 10^8
GOx/ITO/before sintering	0.5 (0.020)	13.5 (0.50)	246.0 (3.2)	8.9 (1.3) × 10^3
GOx/ITO/after sintering	0.2 (0.001)	1680.0 (41.0)	4.2 (0.1)	6.3 (6.3) × 10^1

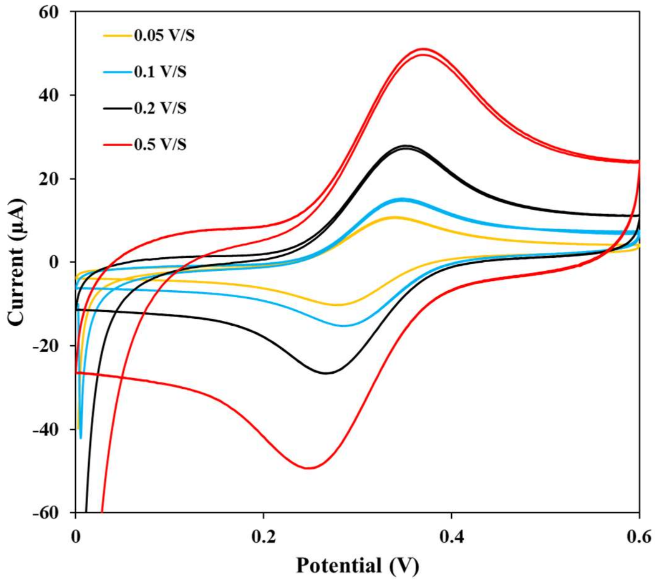

Figure S3. CV curves of 10 nM dsCGG-8 at different scan rates.
